# School Based Sex Education and HIV Prevention in Low- and Middle-Income Countries: A Systematic Review and Meta-Analysis

**DOI:** 10.1371/journal.pone.0089692

**Published:** 2014-03-04

**Authors:** Virginia A. Fonner, Kevin S. Armstrong, Caitlin E. Kennedy, Kevin R. O'Reilly, Michael D. Sweat

**Affiliations:** 1 Johns Hopkins Bloomberg School of Public Health, International Health Department, Baltimore, Maryland, United States of America; 2 Medical University of South Carolina, Department of Psychiatry and Behavioral Sciences, Charleston, South Carolina, United States of America; Vanderbilt University, United States of America

## Abstract

**Objectives:**

School-based sex education is a cornerstone of HIV prevention for adolescents who continue to bear a disproportionally high HIV burden globally. We systematically reviewed and meta-analyzed the existing evidence for school-based sex education interventions in low- and middle-income countries to determine the efficacy of these interventions in changing HIV-related knowledge and risk behaviors.

**Methods:**

We searched five electronic databases, PubMed, Embase, PsycInfo, CINAHL, and Sociological Abstracts, for eligible articles. We also conducted hand-searching of key journals and secondary reference searching of included articles to identify potential studies. Intervention effects were synthesized through random effects meta-analysis for five outcomes: HIV knowledge, self-efficacy, sexual debut, condom use, and number of sexual partners.

**Results:**

Of 6191 unique citations initially identified, 64 studies in 63 articles were included in the review. Nine interventions either focused exclusively on abstinence (abstinence-only) or emphasized abstinence (abstinence-plus), whereas the remaining 55 interventions provided comprehensive sex education. Thirty-three studies were able to be meta-analyzed across five HIV-related outcomes. Results from meta-analysis demonstrate that school-based sex education is an effective strategy for reducing HIV-related risk. Students who received school-based sex education interventions had significantly greater HIV knowledge (Hedges *g* = 0.63, 95% Confidence Interval (CI): 0.49–0.78, p<0.001), self-efficacy related to refusing sex or condom use (Hedges *g* = 0.25, 95% CI: 0.14–0.36, p<0.001), condom use (OR = 1.34, 95% CI: 1.18–1.52, p<0.001), fewer sexual partners (OR = 0.75, 95% CI:0.67–0.84, p<0.001) and less initiation of first sex during follow-up (OR = 0.66, 95% CI: 0.54–0.83, p<0.001).

**Conclusions:**

The paucity of abstinence-only or abstinence-plus interventions identified during the review made comparisons between the predominant comprehensive and less common abstinence-focused programs difficult. Comprehensive school-based sex education interventions adapted from effective programs and those involving a range of school-based and community-based components had the largest impact on changing HIV-related behaviors.

## Introduction

Worldwide, young people aged 15–24 accounted for almost half of all new HIV infections among individuals aged 15 and older in 2010 [1]. School-based sex education is an intervention that has been promoted to increase HIV-related knowledge and shape safer sexual behaviors to help prevent new infections among this vulnerable group. As sexual debut is common in adolescence, so are the associated risks of engaging in transactional sex, having multiple concurrent partnerships, and experiencing sexual violence and coercion, all of which increase HIV-related risk [2]. School-based interventions are logistically well-suited to educate youth about sexual activity given their ability to reach large numbers of young people in an environment already equipped to facilitate educational lessons and group learning [3].

Contentious debates have raged in the past decade regarding whether abstinence-only or comprehensive sexual education interventions are effective and appropriate. Abstinence-only interventions promote delaying sex until marriage with little to no information provided about contraceptives or condom use, whereas comprehensive sexual education provides information on abstinence as well as information on how to engage in safer sex and prevent pregnancies and sexually transmitted infections (STIs). In the 1990s various groups in the United States invested in abstinence-only education, and with the creation of the President's Emergency Plan for AIDS Relief (PEPFAR) in 2004, money was earmarked for “ABC” programs (abstain, be faithful, use condoms), with a heavy emphasis on the “A” component, to be implemented in low- and middle-income countries most impacted by HIV [4]. Critics of abstinence-only education claim that it violates human rights by withholding potentially life-saving information from people about other means to protect themselves from HIV, such as condom use [5]. Others argue that abstinence is only a viable option for those who are able to choose when, how, and with whom to have sex, which is not always the case for many young women [6]. Additionally, promoting abstinence until marriage excludes gay children and adolescents who have no option for marriage in most countries. As an alternative, comprehensive school-based sex education programs present participants with all prevention options, including condom use and partner reduction. Abstinence-plus interventions present prevention options as hierarchical with abstinence being presented as the only strategy that completely eliminates HIV/STI risk.

Previous research has been conducted on the effectiveness of youth-oriented HIV prevention and sex education interventions in school settings. A review of 35 school-based sex education programs by Kirby and Coyle [7] found that abstinence based programs had no significant effect on delaying sexual debut, while some comprehensive programs were effective in reducing certain sexual risk behaviors. Gallant and Maticka-Tyndale [3] reviewed 11 school-based HIV education programs in Africa and concluded that most studies had an effect on either increasing HIV-related knowledge or changing attitudes or behaviors relating to sexual risk. Paul-Ebhohimhen et al. [8] systematically reviewed 10 school-based sex education studies implemented in sub-Saharan Africa and noted that interventions were more likely to report changes in knowledge as opposed to changes in sexual behavior. Speizer et al. [9] reviewed 41 adolescent reproductive health interventions in developing countries, including 22 based in schools, and found that the majority of school-based interventions (17/21) demonstrated improved HIV-related knowledge. Chin et al. [10] conducted parallel systematic reviews and meta-analyses of comprehensive and abstinence-only educational interventions and found that comprehensive sex education programs significantly reduced HIV, STI, and unintended pregnancies, but results for the abstinence-only review were inconclusive.

However, few reviews have attempted to quantitatively synthesize the effects of school-based interventions on HIV-related risk behaviors across studies, and no review to date has attempted to compare the effectiveness of abstinence-only or abstinence plus interventions with comprehensive sex education in low- and middle-income countries. Therefore, the current review seeks to address this gap by conducting a systematic review and meta-analysis on the efficacy of school-based sex education interventions, including abstinence-only/abstinence-plus and comprehensive sex education programs, in changing HIV-related knowledge and risk behaviors in low- and middle-income countries. This review sought to answer the following research question: Does participating in school-based sex education vs. not participating in school-based sex education reduce HIV-related risk behaviors among youth in low- and middle-income countries?

## Methods

This review is part of a large systematic review and meta-analysis project, called The Evidence Project, which is a joint collaboration between investigators at the Medical University of South Carolina and the Johns Hopkins Bloomberg School of Public Health. The Evidence Project reviews the efficacy of behavioral HIV prevention interventions in low- and middle-income countries. Other reviews published with this project include topics such as voluntary counseling and testing [11], [12], provider-initiated testing and counseling [13], condom social marketing [14], behavioral interventions for people living with HIV [15], peer education [16], psychosocial support [17], mass media [18], and treatment as prevention [19]. This review used standardized data abstraction forms and procedures that have been employed in all reviews published as part of The Evidence Project, although no standalone protocol has been published specifically for this review. Additionally, we followed standard systematic review and meta-analysis procedures set forth in the Preferred Reporting Items for Systematic Reviews and Meta-Analyses (PRISMA) guidelines [20].

### Definition and Inclusion Criteria

School-based sex education was defined as programs designed to encourage sexual risk reduction strategies for HIV prevention delivered in school settings. This definition allowed for the inclusion of abstinence-only, abstinence-plus, and comprehensive sex education programs. Studies included in the review had to meet the following criteria: conducted in a low- or middle-income country as defined by the World Bank [21]; published in a peer-reviewed journal from January 1, 1990 to June 16, 2010; presented results of pre-post or multi-arm experimental design and analysis of outcome(s) of interest; and involved an HIV prevention intervention administered in a school setting that encouraged one or more sexual risk reduction strategies, including abstinence, condom use, or partner reduction.

There were no restrictions on language; eligible non-English articles were translated by consultants fluent in English and the language in which the article was written. Participant age was also not restricted. Therefore, studies across a variety of educational settings, from primary schools through college and vocational schools, were included. Additionally, in order to include as many studies as possible, a wide range of study designs were eligible for inclusion: randomized controlled trials (both individual and cluster-randomized, i.e., school or classroom), non-randomized controlled trials, prospective or retrospective cohorts, time-series, before-after, case-control, cross-sectional, and serial cross-sectional studies.

### Search strategy

Our search strategy involved three methods. First, five electronic databases, including PubMed, PsycInfo, EMBASE, CINAHL, and Sociological Abstracts, were searched using a combination of terms for sex education, schools/youth, and HIV or AIDS (full list available from the authors upon request). The search was limited to a date range of January 1, 1990 to June 16, 2010. We also searched the table of contents of *AIDS*, *AIDS Care*, *AIDS and Behavior*, and *AIDS Education and Prevention* for relevant citations. Finally, we searched the reference lists of all included studies for additional eligible studies. This process was iterative and continued until no additional studies were identified.

Trained research assistants conducted an initial screening of all citations and excluded studies clearly not relevant to school-based sex education. Two senior study staff members then independently screened all remaining citations and categorized studies as eligible for inclusion, not eligible for inclusion, or questionable. Discrepancies in categorization were resolved through consensus. Full article texts were obtained and discussed by senior researchers to ascertain eligibility if questionable. Articles were retained and included as background studies if they failed to meet the inclusion criteria but still contained information relevant to school-based sex education in low- and middle-income countries, including prior reviews, cost-effectiveness analyses, and qualitative studies.

### Data Abstraction

The following data were abstracted from each eligible study using standardized forms: location, year(s) of study implementation, study setting, study population, sample size, study design, sampling frame and sampling methods, description of the intervention, composition of intervention and control groups (if applicable), length of follow-up, description of outcomes, effect sizes, confidence intervals, statistical tests employed, and study limitations. Two trained research assistants independently abstracted data from each study; any discrepancies were resolved through consensus. Data were double entered into EpiData version 3.1 (EpiData Association, Odense, Denmark) and later exported to an SPSS database (IBM SPSS Statistics for Windows, Version 20.0. Armonk, NY).

We also evaluated the methodological rigor of studies to assess risk of bias based on the following criteria: whether the study 1) included a cohort of participants, 2) had a control and experimental/intervention group, 3) compared baseline demographic equivalence of control and intervention groups, 4) compared outcome measures between control and intervention groups at baseline (if applicable), 5) contained both pre- and post-intervention data, 6) randomly selected participants for assessment (i.e. sampling strategy), 7) randomly assigned participants to the intervention, and 8) maintained a follow-up rate of greater than 80%.

### Selection of outcomes

Outcomes were chosen for meta-analysis based on relevance to HIV prevention and frequency in available studies. The five most commonly reported outcomes across studies were: HIV knowledge, condom use, self-efficacy related to HIV prevention (e.g., confidence in refusing sex or confidence in using condoms during sex), initiation of first sex, and number of sexual partners. All outcomes were based on self-report. Studies containing at least one of these outcomes were included in meta-analysis if they met the following criteria:

1. Provided an estimate of effect size and its variance, or provided statistics needed to calculate an effect size and variance. If enough information was not provided to calculate an effect size, study authors were contacted for clarification or additional statistics. If study authors did not provide this information after one month, the study was removed from the analysis.
2. Presented pre-post or multi-arm results comparing either participants who received the intervention to those who did not, or comparing outcomes before and after the intervention. If results of a repeated measures analysis were reported, authors needed to provide the correlation between pre-post measurements or provide enough information to calculate the correlation between measurements. If these statistics were not available, either in publication or after request, and the study was a controlled design, an effect size was generated using post-intervention statistics provided groups were similar at baseline with respect to the outcome of interest and other relevant covariates.
3. Presented an outcome of interest that was measured in such a way as to be comparable to outcomes assessed by other studies. In other words, outcomes needed to be similar enough to synthesize across studies.
4. Presented data based on an individual unit of analysis (studies presenting classroom- or school-level data only were excluded from meta-analysis).

### Meta-analysis

Using standard meta-analytic methods [22], we standardized effect sizes as either Hedges' *g* (for continuous outcomes) or odds ratios (for dichotomous outcomes). For several outcomes, including HIV knowledge, self-efficacy, and number of sexual partners, both continuous and dichotomous effect sizes were combined in meta-analysis. In these instances, Comprehensive Meta-Analysis (CMA) was used to either convert the standard mean difference into an odds ratio when transforming the effect size from continuous to dichotomous or vice versa using methods developed by Hasselblad and Hedges [23]. This transformation assumes that the outcome under study involves an underlying continuous trait with a logistic distribution [24] and that outcomes are measured in relatively similar terms, regardless of whether they are presented dichotomously or continuously. For example, several studies reported number of sexual partners as a dichotomous outcome, such as having two or more partners in the past 6 months, whereas others reported a mean number of partners. The same logic holds for outcomes such as knowledge, where some studies presented knowledge outcomes on a continuous scale whereas others created a cut-off for “high” and “low” knowledge scores and presented results as a proportion. Combining both dichotomous and continuous effect sizes allowed us to utilize all available data.

CMA V.2.2 was used for all analyses [25]. Random effects models were used as included studies contained considerable heterogeneity of effects, and the purpose of the analysis was to generate inferences beyond the set of included studies [26].

When possible, data were analyzed in several ways per outcome. Stratifications by age, gender, instructor (e.g., teacher, peer, or health care professional), intervention type (abstinence vs. comprehensive sex education), and length of follow-up were made when three or more studies could be retained per category. Additionally, when possible, we investigated the role of certain characteristics of the data itself, including comparing differences between continuous and dichotomous effect sizes and whether the effect size was based on data collected pre-post intervention or post-only. Mixed effects meta-regression techniques were used to compare effect sizes across strata when possible. The I^2^ statistic and its confidence interval were calculated for each meta-analysis to describe inconsistencies in effect sizes across studies [24], [27]. When possible adjusted effect sizes were used in the pooled analyses; however, outcomes were most frequently reported in unadjusted terms, thus the analyses contain both adjusted and unadjusted effect sizes. Potential bias across studies, such as publication bias and selective reporting, was assessed for the HIV-related knowledge outcome by constructing a funnel plot. Funnel plots were not constructed for the remaining meta-analyses because there were too few studies to interpret the dispersion of effect sizes across the range of standard errors.

## Results

### Description of studies

**Figure 1:**
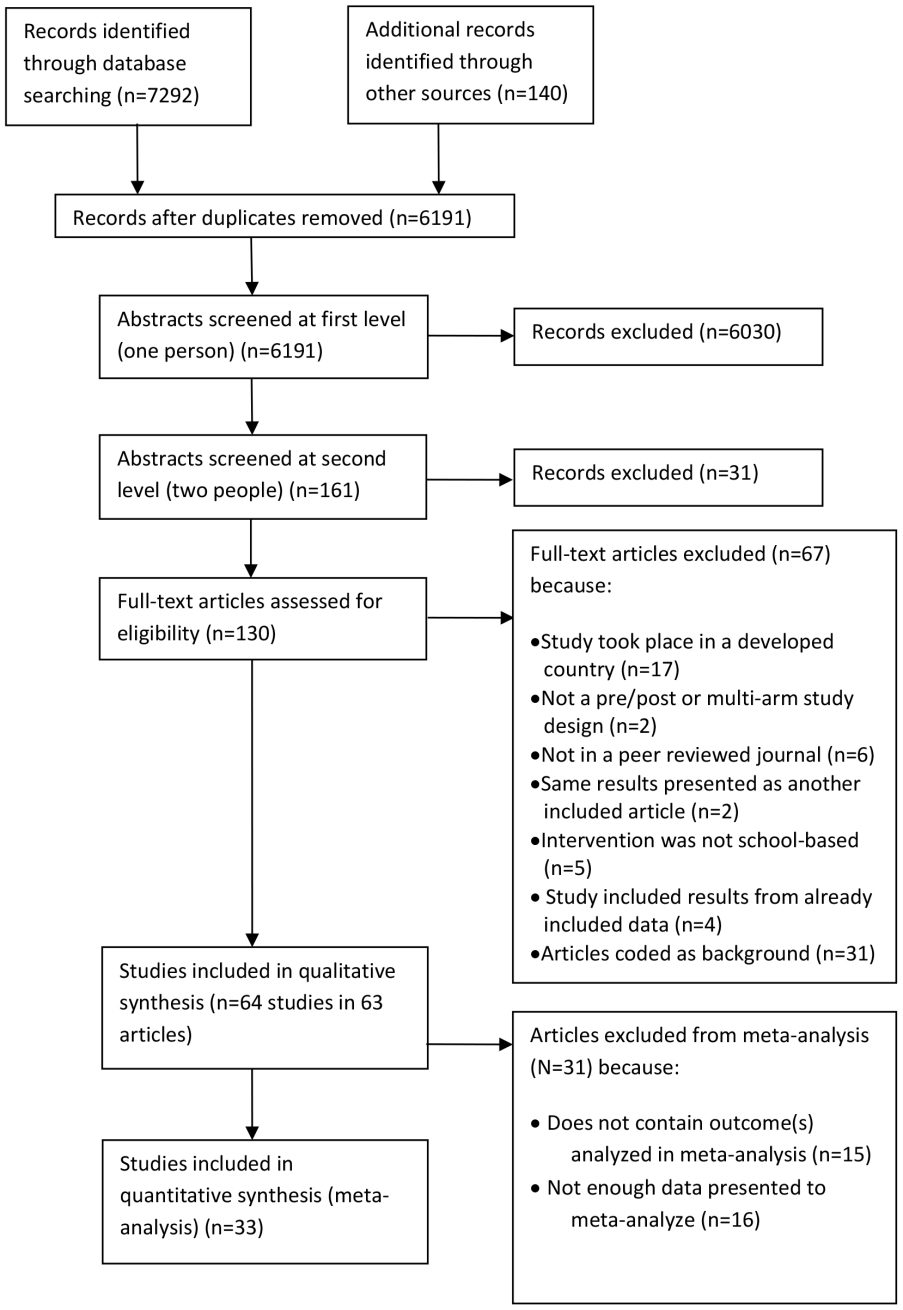
Disposition of citations during the search and screening process.

Of 6191 studies initially identified, 64 studies in 63 articles met the inclusion criteria for this review (Figure 1). In five cases, more than one article presented data from the same study [28]–[39]. If articles from the same study presented different outcomes or follow-up times, both articles were retained and included in the review as one study [30], [31], [37], [38]. If both articles presented similar data, such as by providing an update with longer follow-up, the most recent article or the article with the largest sample size was chosen for inclusion [28], [33], [36], [39].

Table 1 provides descriptions of included studies. The majority of studies took place in sub-Saharan Africa (n = 29, 45.3%). Studies also took place in East Asia/Pacific (n = 15, 23.4%), Europe/Central Asia (n = 2, 3.1%), Latin American/Caribbean (n = 16, 25.0%), and South Asia (n = 4, 6.3%). The most commonly used study design was a group randomized trial (n = 21), with schools or classrooms as the unit of randomization. Other study designs included individual randomized controlled trials (n = 4), before-after studies (n = 14), non-randomized individual trials (n = 2), non-randomized group trials (n = 12), serial cross-sectional studies (n = 4), cross-sectional studies (n = 5), and two studies utilized a study design classified as “other” which involved a hybrid of eligible study designs.

**Table 1:** Description of studies included in the review.

Study	Setting	Population Age Characteristics (in years)	Intervention Description	Study Design	n (Baseline)	Participant Selection	Outcomes Used in Meta-Analysis
Agha et al., 2004 [29]	Zambia (Central, Copperbelt, and Lusaka provinces)	Range: 14–23	Peer sexual health intervention, including an open discussion about abstinence, condom use, and risks of acquiring STIs/HIV, drama skits performed by peer educators; and distribution of leaflets about STIs.	Group randomized trial	n = 416 (I = 254; C = 162)	Random	
Antunes et al., 1997 [99]	Brazil (Sao Paulo)	Mean: 19.9	Trained teachers facilitated student discussion of AIDS-related knowledge. Community building was fostered through AIDS training courses for teachers, peer support programs, and public events.	Group randomized trial with delayed control	n = 394 (I = 150; C = 154)	Random	
Aplasca et al., 1995 [56]	Philippines (Manila)	Mean: (I): 14.7, (C): 14.9	AIDS education curriculum focused on delaying sexual activity until adulthood and/or within marriage, although condom use was also discussed.	Group randomized trial	n = 845 (I = 438; C = 407)	Random	
Caceres et al., 1994 [71]	Peru (Lima)	Mean: 15.5; Range: 11–21	Aimed at empowering adolescents regarding their sexuality, improving knowledge and attitudes, and developing skills and prevention-oriented behavioral intentions through various activities.	Group randomized trial	n = 1213 (I = 604; C = 609)	Non-random	
Cai et al., 2008 [100]	China (Shanghai)	Mean: 17.22 (SD: 0.89)	Intervention description not reported. Authors refer to intervention as school based and peer led education program.	Individual randomized trial	n = 1950 (I = 968; C = 982)	Random	
Cartagena et al., 2006 [73]	Mongolia (Ulaanbaatar, South and Middle Gobi Aimag, Nalaikh, Tuv Aimag, Baganuur Bolovsrol)	%<17: (I): 20.2%, (C): 11.9% %≥17: (I): 79.8%, (C): 88.1%	Peer educators received three day training on reproductive system anatomy; HIV and STI knowledge, including methods of transmission and prevention; condom demonstrations; discussion of communication, and more general life skills about good decisions and risk taking.	Cross sectional study	n = 647 (3 years post-intervention) (I = 320; C = 327)	Mixed	Condom use
Cheng et al., 2008 [74]	China (Shangcai County, Henan Province)	Range: 14–18	Life-planning skills program utilizing participatory training methods, combining information education with skills building.	Non-randomized group trial	n = 1132 (I = 717; C = 457)	Non-random	
Chhabra et al., 2008 [79]	India (Mumbai)	Range: 13–15	HIV prevention program built on specific cultural, linguistic, and community-specific characteristics. Trainers conducted the program with eighth graders.	Group randomized trial	n = 1846 (I = 946; C = 900)	Random	HIV knowledge
Daboer et al., 2008 [90]	Nigeria (Jos North and South Local Government Area, Plateau State)	Mean: (I): 17.6, (C): 17.8	Health education covering the meaning of HIV/AIDS, HIV/AIDS symptoms, activities considered high risk for HIV/AIDS, HIV/AIDS prevention and control, and life skills.	Group randomized trial	n = 1246 (I = 620; C = 626)	Random	
Dalrymple et al. 1993 [61]	South Africa (Zululand)	Not Reported	Education about AIDS and AIDS prevention in three phases: 1) theatre programme; 2) drama workshops/lessons; and 3) students present songs, poems, speeches and plays to the community and parents on AIDS.	Before-after study	n = 72	Not reported	HIV knowledge
Diaz et al., 2005a–c [52]	Brazil (a. Salvador; b. Rio de Janeiro; c. Belo Horizonte)	% 10–14: a, b, c (I): 55.2%, 55.7%, 60.3% (C): 44.4%, 60.6%, 65.5%; % 15–19: a, b, c (I): 44.8%, 44.3%, 39.7% (C): 55.6%, 39.4%, 34.5%	Sex education program, implemented by local NGOs in partnership with the public school and health systems, focused on sexuality, gender, and citizenship as crosscutting themes, as well as an expanded concept of sexuality, changing the common comprehension of sexuality as intercourse.	Cross sectional study	Not reported	Non-random	HIV knowledge; Condom use
Doyle et al., 2010 [30] and Ross et al, 2007 [37]	Tanzania (Mwanza Region)	Median: (Male): 22 (Female): 21	In-school programs in primary school years 5–7, youth-friendly health services, commubity-based condom promotion and distribution, and community-mobilization activities.	Group randomized trial	n = 13,814 (Doyle) (I = 7089; C = 6731) n = 9645 (Ross) (I = 4870; C = 4775)	Non-random	HIV knowledge; Condom use; initiation of sex; number of sex partners
Enah et al., 2010 [40]	Cameroon (Buea)	Mean: 10.5; Range: 10–12	One-day intensive education program to influence attitudes, control beliefs and revise behavioral norms about delaying sex, and help students prepare responses to use when faced with pressures to have sex.	Before-after study	n = 60	Non-random	
Fawole et al., 1999 [86]	Nigeria (Ibadjan)	Mean: (I): 17.6, (C): 17.8	Comprehensive health education curriculum using health education tools such as lectures, film shows, role-plays, stories, songs, debates and essays.	Non-randomized group trial	n = 450 (I = 233; C = 217)	Random	HIV knowledge; Condom use
Fiscian et al., 2009 [41]	Ghana (Nsawam)	Range: 10–14	Abstinence-based HIV prevention education program covering HIV transmission and prevention, relationships with sugar daddies, approaches for condom and sex negotiation, expressing feelings, benefits of abstinence, and income generation skills.	Before-after study	n = 61	Non-random	HIV knowledge
Fitzgerald et al., 1999 [31] and Stanton et al., 1997 [38]	Namibia (Caprivi and Omusati regions)	Mean: 17; Range: 15–18	Sexual health education curriculum focused on basic facts about reproductive biology and HIV/AIDS, other risk behaviors communication skills across gender and age differences, , and a framework for decision-making.	Individual randomized trial	n = 515 (I = 262; C = 253)	Random	HIV knowledge; Condom use; initiation of sex; number of sex partners; self-efficacy
Gallegos et al., 2008 [101]	Mexico (Monterrey)	Mean: 15.2	Program focused on reducing the risk of HIV/AIDS which also addressed condom and contraceptive use.	Randomized individual	n = 829 (I = 454; C = 375)	Non-random	
Gao et. al., 2001 [48]	China (Beijing, Shanghai)	Not Reported	Senior medical students were trained to teach their junior peers about HIV/AIDS, STDs and safer sex.	Group randomized trial	Not reported	Random	
Givaudan et al., 2008 [81]	Mexico (Toluca)	Mean: 15.97	Comprehensive AIDS and sexual health education curriculum, including activities for students to practice the skills they learned.	Group randomized trial	n = 2,064 (Ns by study group not reported)	Random	HIV knowledge; self-efficacy
Halpern et al., 2008a-Kenya [64] and 2008b Brazil [64]	Kenya (Nairobi) Brazil (Rio de Janeiro)	Mean: (Nairobi): 16.5, (Rio): 14.71	Easy access to web-based reproductive health information, combined with intellectual “priming” about reproductive health topics.	Non-randomized group trial	n = 1530 (Ns by study group not reported)	Non-random	
Harvey et. al, 2000 [62]	South Africa (KwaZulu Natal)	Median: 17.6; Range: 13–29	A play about HIV performed by teachers and nurses, followed by running drama workshops for students, and culminated with a celebratory day on HIV/AIDS with drama, song.	Group randomized trial	n = 1080 (Ns by study group not reported)	Non-random	
Iurcovich et al., 1998 [72]	Argentina (Buenos Aires)	Mean: 14.7	Workshop divided into three sections: HIV-related information, group exercises, and discussion time.	Serial cross-sectional study	n = 132	Non-random	
James et al., 2006 [53]	South Africa (Pietermaritzburg region of KwaZulu-Natal)	Mean: 15.5; Range: 12–21	Program focused on HIV/AIDS knowledge, attitudes towards condom use and people living with HIV/AIDS, gender norms and perceptions about sexual behavior.	Randomized group trial	n = 1141 (I = 628; C = 513)	Random	HIV knowledge
Karnell et al., 2006 [77]	South Africa (Pietermaritzburg region of KwaZulu-Natal)	Median: 16	HIV prevention curriculum to impart key HIV and alcohol related facts.	Randomized group trial	n = 661 (I = 325; C = 336)	Random	
Kinsler et al., 2004 [82]	Belize (Belize City)	Range: 13–17	HIV prevention sessions on impacting attitudes towards condoms, peer norms regarding sex and condoms, communication skills and intention to use condoms.	Non-randomized trial	n = 150 (I = 75; C = 75)	Non-random	HIV knowledge; self-efficacy
Kinsman et. al, 2001 [65]	Uganda (Masaka District)	Mean: (I): 14.3, (C): 14.5; Range: (I): 10–24, (C): 10–25	Teacher-led health education curriculum focused on 4 units: basic information about HIV/AIDS; responsible behavior (delaying sex); responsible behavior (protected sex); and caring for people with AIDS.	Group randomized trial	n = 31 schools (I = 20; C = 11)	Random	
Klepp et al., 1997 [33]	Tanzania (Arusha, Kilimanjaro)	Mean: 13.6 (SD: 1.3)	AIDS education program to strengthen pupils' intentions not to engage in sex in the near future and communication between parents and community members on HIV/AIDS issues.	Group randomized control	n = 814 (I = 258; C = 556)	Random	HIV knowledge; initiation of sex
Kuhn et al., 1994 [54]	South Africa (Cape Town)	Mean: 18; Range: 12–30	Educational methods including structured information sessions on AIDS, open discussions about AIDS, and integration of AIDS content into the language curriculum.	Non-randomized group trial	n = 567 (I = 231; C = 336)	Not reported	HIV knowledge
Kryrychenko et al., 2006 [83]	Ukraine (Vinnitsa)	Range: 15–16	HIV/AIDS education intervention on HIV biology, transmission, prevention, condom-negotiation skills and dangers of drug use.	Non-randomized group trial	n = 200 (I = 100; C = 100)	Random	HIV knowledge; self-efficacy
Li et al., 2008	China (Nanjing)	Mean: 20.0	Culturally adapted, social cognitive-theory-based, HIV risk reduction program in college.	Randomized group trial	n = 374 (I = 186; C = 194)	Random	HIV knowledge; self-efficacy
Li et al., 2010 [84]	China (Shanghai)	Mean: 14.20 (SD: 1.19)	Peer-led HIV/AIDS intervention to increase HIV/AIDS knowledge, STD; prevention measures; improving self-efficacy, and eliminating prejudice and stigmatization toward people living with HIV/AIDS.	Non-randomized individual trial	n = 2237 (I = 1140; C = 1097)	Non-random	HIV knowledge; self-efficacy
Lou et al., 2006 [36]	China (Shanghai)	%<17: (I): 43.6%, (C): 48.4%; %≥17: (I): 56.4%, (C): 51.7%	Website specially designed for study, comprised 8 sections: puberty; sex psychology and morality; STDs; HIV/AIDS; reproduction and contraception; harm of premarital sex and abortion; self-protections against sexual harassment; hazard of smoking/drug abusing; reproductive health; information and consultation for contraception.	Non-randomized group trial	n = 1337 (I = 624; C = 713)	Non-random	
Low et al., 2004 [44]	Malaysia (Kuala Lumpur)	Median: 24.20; Range: 21–38	Course by faculty of medicine on reproductive health, including family planning and pregnancy, STDs, HIV/AIDS, relationship and marriage, sexual dysfunctions, alternative sexual behaviors, sexuality and disability, physiology of sex, gender issues in sexuality and violence against women.	Before-after study	n = 85	Non-random	
Magnani et al., 2005 [78]	South Africa (KwaZulu-Natal Province)	Range: 14–22	Life skills education program on: drugs and alcohol; STI transmission, symptoms, and prevention; HIV transmission and prevention; violence and sexual abuse; relationships: negotiation and assertiveness; contraception; reproductive biology; and self-esteem.	Dose-response	Survey 1 n = 3052 Survey 2 n = 4185	Non-random	
Martinez-Donate al., 2004 [85]	Mexico (Tijuana)	Mean: 17.6 (SD: 1.5)	HIV prevention workshop addressing: 1) HIV-related attitudes and risk behaviors, 2) effect of AIDS on health and family, 3) facts about HIV/AIDS, transmission and prevention, 4) HIV transmission, 5) living with HIV, 6) myths and facts about HIV/AIDS, 7) condom use and negotiations skills. A free condom kiosk at schools was provided to one intervention group.	Group randomized trial	n = 320 (I = 198; C = 122)	Not reported	Condom use; initiation of sex; self-efficacy
Martiniuk et al., 2003 [69]	Belize (Belize City)	Mean: (I): 15.6, (C): 15.5	Scripted sexuality education intervention aimed to provide a framework for adolescents on how to make responsible decisions within relationships.	Group randomized trial	n = 468 (I = 197; C = 271)	Not reported	
Maticka-Tyndale et al. 2010 [68]	Kenya (Nyanza Province)	Mean: 14.1; Range: 11–17	Teachers were trained to train colleagues in their home schools, to integrate HIV educaiton throughout classroom subjects, and to provide guidance and counseling on HIV-related topics. Teachers used anonymous quetsion boxes, school health clubs, and other school activities, like drama, to facilitate learning about HIV.	Serial cross-sectional study	n = 953	Mixed	HIV knowledge; self-efficacy
Milleliri et al., 2003 [63]	Gabon (Libreville, Lamborene)	Mean: 1	A comic book with scenarios aiming to demystify condoms and reduce HIV transmission through behavior change, and a class talk on HIV/AIDS (definition, epidemiology, transmission, and prevention).	Before-after study	n = 964	Random	
Miller et al., 2008 [45]	Kenya (Nairobi – Kenyatta University Main Campus)	Not reported	Students who were considered “cool” by campus standards were invited to participate in high-energy 3-month HIV training programs. Other activities included sexual purity pledge cards and t-shirts advocating general sexual responsibility, and small and large group messages on faithfulness and condom use, alternated with calls to save sex until marriage. Multiple VCT clinics were conducted, and an annual HIV testing day was organized.	Serial cross-sectional study	n = 632	Random	
Muodawafa et al., 1995 [49]	Zimbabwe (Masvingo Province)	Not reported	Curriculum included education about transmission, prevention, psychosocial issues of AIDS and STDs, responsible sexual behavior, and problem-solving and decision-making strategies.	Non-randomized group trial	n = 285 (I = 141; C = 144)	Not reported	
Norr et al., 2007 [50]	Malawi (Lilongwe and Blantyre)	%>30: 44%	Sessions with information on condoms, HIV prevention, transmission, stigma, sexuality, STDs, and safe sex negotiation.	Before-after study	n = 382	Non-random	
Okonofua et al., 2003 [66]	Nigeria (Edo State)	Range: 12–25	Community participation, peer education, public lectures, health clubs in the schools, and training of STD treatment providers, including those with no formal training.	Group randomized trial	n = 1896 (I = 643; C = 1253)	Non-random	Condom use
Perez et al., 2003 [67]	Columbia (Santa Fe de Bogota, Cali, and Bucaramanga)	Median: (Santa Fe de Bogota): 16; (Bucaramanga.): 15; (Cali): 16	HIV/AIDS education intervention facilitated by peer educators and teachers aimed to teach appropriate condom use, raise self-esteem and introduce/improve negotiation skills among sexually active youth.	Before-after study	n = 111	Random	
Pick et al., 2007 [51]	Mexico (Hidalgo and Campeche States)	Range: 9–12	Life skills program focused on self-esteems, problem-solving, communication, and on health care issues, including personal hygiene, sexuality and nutrition.	Group randomized trial	n = 1581 (Ns by study group not reported)	Random	
Rusakaniko et al.,1997 [102]	Zimbabwe (Mashonaland Central)	Mean: 13.5 (SD: 1.3)	Health education intervention on HIV/AIDS, reproductive biology, preventing unwanted pregnancy, STDs and family planning.	Group randomized trial	n = 1,689 (I = 1159; C = 530)	Random	HIV knowledge
Saksena et al., 2003 [57]	India (Bangalore)	Range: 13–15	Course on human sexuality covering the reproductive system and anatomy, physiology and puberty changes; emotional aspects of adolescence and dangers to avoid; HIV/AIDS, and contraception.	Before-after study	n = 392	Not Reported	
Shen et al., 2008 [75]	China (Shanghai)	Mean: 17.4; Range: 15–19.9	Program to increase knowledge about reproductive health and contraception, HIV transmission routes, prevention measures and condom demonstrations, STDs, relationships between HIV/AIDS and STD, STD transmission and prevention measures.	Before-after study	n = 1910	Non-random	
Shuey et al., 1999 [58]	Uganda (Soroti district)	Mean: (I): 14, (C): 13.8; Range: (I): 10–18, (C): 9–22	Health education program including meetings of parents, teachers and community leaders to discuss health/sex education issues, training for senior women tutors and science teachers to improve skills as health educators, and training at the local teachers' training colleges on curriculum, forming student health clubs, and competitions in plays, essays, poems and songs on health issues.	Group randomized trial with serial cross-sectional analysis	n = 400 (I = 287; C = 113)	Random	Number of sex partners
Smith et al., 2008 [89]	South Africa (Cape Town)	Mean: 14.0	Sexual education cirriculum as part of life orientation course including social-emotional skills, substance abuse and risk education.	Non-randomized group trial	n = 2,176 (I = 901; C = 1275)	Non-random	
Singh, 2003 [42]	India (city not reported)	Range: 18–22	Three sessions, each focusing on information, motivation, and behavioral skills, respectively.	Non-randomized group trial	n = 200 (I = 100; C = 100)	Non-random	HIV knowledge
Srisuphun et al., 2002 [80]	Thailand (Chiang Mai)	Not reported, (7–12 grade students)	Teachers trained in participatory learning for life skills development to train school-aged peer leaders in skills development, and to conduct school-wide activities on STD/HIV information, negotiation skills, safer sex strategies, and drug use prevention.	Before-after study	n = 414	Non-random	
Thakor et al., 2000 [76]	India (Surat City)	Range: 15–18	Lectures followed by discussion and question-answer sessions. Topics included: anatomy and physiology, STDs and prevention, myths about sex/sexual behavior, and conception/contraception.	Before-after study	n = 189	Non-random	
Thato et al., 2008 [59]	Thailand (Bangkok)	Range: 13–18	Sex education program focused on sexual behavior and condom use, refusing sex, STI, HIV/AIDs, and pregnancy knowledge. No sex before marriage was emphasized.	Non-randomized individual trial	n = 522 (I = 261; C = 261)	Mixed	HIV knowledge; Condom use
Torabi et al., 2000 [70]	Russia (St. Petersburg)	%≤13: 16%; % 14: 51%; %≥15: 33%	Viewing a two-hour video tape focused on prevention of AIDS by using lecture, illustration, and question- and answer techniques covering the nature of the disease, transmission, and prevention.	Non-randomized group trial	n = 1,124 (Ns by study group not reported)	Not reported	
Visser et. al, 1996 [55]	South Africa (urban and rural areas)	% 12–15: 35%; % 16–18: 57%; % 19–25: 6%	AIDS and lifestyle education programme for teenagers aimed to convey accurate information about HIV/AIDS and to provide students with life skills to to negotiate with peers, to make decisions and to establish behavior patterns that prevent health risks.	Before-after study	n = 337	Mixed	HIV knowledge
Visser et al., 2005 [103]	South Africa (Gauteng Province)	% 14–16: 56%; % 17–19: 44%	Life skills and HIV/education program as part of national curriculum aiming to increase knowledge/skills for healthy relationships, effective communication and responsible decision-making, and to promote positive and responsible attitudes towards people with HIV/AIDS.	Before-after study	n = 667	Not reported	HIV knowledge
Visser et al., 2007 [87]	South Africa (Tshwane)	Range: 13–20	Peer educators raised awareness and knowledge of HIV through school activities; mobilised involvement in promotion of healthy behavior; created context to discuss sexual relationships, gender issues; were available for informal conversation, support and guidance; and being role models.	Serial cross-sectional study	n = 1982	Random	Condom use; number of sex partners
Walker et al., 2006 [88]	Mexico (Morelos)	Mean: (I Male): 16.9, (I Female): 16.8, (C Male): 16.8, (C Female): 16.7	Curriculum based on teaching life skills with half class time focused on consequences of unprotcted sex and how to avoid it. Other classes dealt with social pressures influencing sexual behavior and provided practice in communciation, negotiation, and refusal skills.	Randomized group trial	n = 10,954 (Ns by study group not reported)	Random	HIV knowledge
Wang et al., 2005 [60]	China (Shanghai)	Mean: 18.5	Sex education program gave information and services on abstinence, sexuality, contraception and HIV/AIDS prevention.	Non-randomized group trial	n = 873 (in-school youth) (I = 497; C = 376)	Non-random	Initiation of sex
Ye et al., 2009 [39] and Huang et al., 2008 [32]	China (Fujian Province Sanming City)	Mean: 17.5	Peer-led HIV/AIDS intervention emphasizing basic knowledge and attitude towards HIV/AIDS, STDs, pregnancy and related skills. The intervention was integrated into the regular school health education.	Randomized individual trial	n = 893 (Ye) (I = 420; C = 473) n = 3068 (Huang) (I = 981; C = 2087)	Random	HIV knowledge
Zheng et al., 2002 [47]	China (Changsha)	Range: 17–22	Health education on sexual behavior, sexual hygiene, fundamentals of HIV/AIDS and other STDs, prevention measures, and attitudes towards AIDS patients.	Before-after study	n = 218	Not reported	

*Note.* I = Intervention and C = Control/Comparison Group.

Table 2 presents the methodological rigor assessment for included studies. Regarding methodological rigor, forty-seven studies used a control group, but only 18 studies reported whether intervention and control groups were equivalent on socio-economic variables at baseline, and only 14 reported whether intervention and control groups were equivalent on outcome measures at baseline.

**Table 2:** Methodological rigor of included studies.

Study	Cohort	Control or comparison group	Pre/Post intervention data	Random assignment of participants to intervention	Random selection of participants for assessment	Follow-up rate of 80% or more	Comparison groups equivalent on socio-demographics	Comparison groups equivalent at baseline on outcome measure
Agha et al., 2004	yes	yes	yes	yes	no	yes	no	no
Antunes et al., 1997	yes	yes	yes	yes	yes	no	no	nr
Aplasca et al., 1995	yes	yes	yes	yes	no	yes	no	nr
Caceres et al., 1994	yes	yes	yes	yes	no	no	nr	yes
Cai et al., 2008	yes	yes	yes	yes	no	yes	yes	yes
Cartagena et al., 2006	no	yes	no	no	yes	n/a	n/a	n/a
Chhabra et al., 2008	yes	yes	yes	yes	yes	yes	nr	nr
Cheng et al., 2008	yes	yes	yes	no	no	yes	yes	yes
Daboer et al., 2008	yes	yes	yes	yes	no	yes	yes	nr
Dalrymple et al. 1993	yes	no	yes	nr	nr	nr	n/a	n/a
Diaz et al., 2005a–c	no	yes	no	no	no	n/a	n/a	n/a
Doyle et al., 2010	no	yes	no	yes	n/a	n/a	nr	n/a
Enah et al., 2010	yes	no	yes	n/a	no	yes	n/a	n/a
Fawole et al., 1999	yes	yes	yes	no	no	yes	yes	yes
Fiscian et al., 2009	yes	no	yes	no	no	yes	n/a	n/a
Fitzgerald et al., 1999	yes	yes	yes	yes	no	yes	yes	no
Gallegos et al., 2008	yes	yes	yes	yes	no	yes	yes	yes
Gao et. al., 2001	yes	yes	yes	yes	nr	nr	nr	yes
Givaudan et al., 2008	yes	yes	yes	yes	no	yes	nr	nr
Halpern et al., 2008a	yes	yes	yes	no	no	no	yes	yes
Halpern et al., 2008b	yes	yes	yes	no	no	no	no	yes
Harvey et. al, 2000	yes	yes	yes	yes	no	no	yes	no
Iurcovich et al., 1998	no	no	yes	n/a	no	n/a	n/a	n/a
James et al., 2009	yes	yes	yes	yes	yes	no	yes^a^	nr
Karnell et al., 2006	yes	yes	yes	yes	yes	yes	yes	no
Kinsler et al., 2004	yes	yes	yes	no	no	yes	yes	yes
Kinsman et. al, 2001	yes	yes	yes	yes	yes	n/a^b^	yes	nr
Klepp et al., 1997	yes	yes	yes	yes	no	no	yes	yes
Kuhn et al., 1994	yes	yes	yes	nr	nr	nr	nr	no
Kryrychenko et al., 2006	yes	yes	yes	no	yes	yes	yes	yes
Li et al., 2008	yes	yes	yes	yes	yes	yes	yes	no
Li et al., 2010	yes	yes	yes	no	yes	yes	no	yes
Lou et al., 2006	yes	yes	yes	no	no	yes	yes	yes
Low et al., 2004	yes	no	yes	no	no	no	n/a	n/a
Magnani et al., 2005	yes	no	no	no	yes	no	n/a	n/a
Martinez-Donate et al., 2004	yes	yes	yes	yes	no	yes	yes	no
Martiniuk et al., 2003	yes	yes	yes	yes	yes	yes	no	nr
Maticka-Tyndale et al., 2010	no	no	yes	no	yes	nr	n/a	n/a
Milleliri et al., 2003	yes	no	yes	n/a	yes	yes	n/a	n/a
Miller et al., 2008	no	no	yes	no	yes	n/a	n/a	n/a
Muodawafa et al., 1995	yes	yes	yes	no	no	yes	nr	nr
Norr et al., 2007	yes	no	yes	n/a	no	yes	n/a	n/a
Okonofua et al., 2003	yes	yes	yes	yes	no	yes	no	no
Perez et al., 2003	yes	no	yes	no	yes	yes	n/a	n/a
Pick et al., 2007	yes	yes	yes	yes	nr	yes	nr	nr
Ross et al., 2007	yes	yes	yes	yes	yes	no	no	yes
Rusakaniko et al.,2007	yes	yes	yes	yes	yes	nr	nr	nr
Saksena et al., 2003	yes	no	yes	no	no	yes	n/a	n/a
Shen et al., 2008	yes	yes	yes	no	no	yes	nr	nr
Shuey et al., yes999	no	yes	yes	yes	yes	no	no	nr
Smith et al., 2008	yes	yes	yes	no	yes	no	no	no
Singh et al., 2003	yes	yes	yes	no	no	yes	nr	yes
Srisuphun et al., 2002	yes	no	yes	no	yes	yes	n/a	n/a
Stanton et al., 1998	yes	yes	yes	yes	no	no	nr	yes
Thakor et al., 2000	yes	no	yes	no	no	yes	n/a	n/a
Thato et al., 2008	yes	yes	yes	no	yes	nr	no	yes
Torabi et al., 2000	yes	yes	yes	no	no	nr	nr	nr
Visser et. al, 1996	yes	no	yes	yes	no	no	n/a	n/a
Visser et al., 2005	yes	no	yes	n/a	no	nr	n/a	n/a
Visser et al., 2007	no	yes	yes	yes	yes	n/a	n/a	n/a
Walker et al., 2006	yes	yes	yes	yes	yes	nr	nr	nr
Wang et al., 2005	yes	yes	yes	no	no	yes	no	nr
Ye et al., 2009	yes	yes	yes	yes^c^	no	yes	yes	no
Zheng et al., 2002	yes	no	yes	n/a	no	yes	n/a	n/a

*Note.*

a Comparison groups were equivalent on all socio-demographic variables except religion.

b Schools are used as the unit of analysis in this paper. Authors do not report whether the individual participant follow-up rate at each of these schools was for greater than 80%.

c Random assignment specified in Huang et al., 2008, not Ye et al., 2009, but articles are reporting on the same study.

n/a: not applicable; nr: not reported.

Most studies included both male and female participants; three studies evaluated school based sex education for girls only [40], [42]. Most studies (n = 56) took place among primary or secondary school students, five studies were implemented among university students [43]–[47], two involved secondary and university students [36], [48], one study involved student nurses [49], and another involved teacher trainees [50]. Ages of study participants ranged from 9, for an intervention among 4^th^ graders in Mexico [51], to 38, for an intervention among university students in Malaysia [44]. Of the 27 studies reporting a mean age of study participants, the average age was 16.5 (SD =  2.7). Many studies included study populations with a wide range of ages. The age range in six studies spanned 10 years or more [52]–[55]. Generally, in studies measuring sexual risk behaviors, only a small portion of students in the study population were sexually active, thus substantially reducing the sample size for these outcomes.

#### Content of interventions

Nine studies either taught abstinence-only or emphasized abstinence or delay of sexual debut over other risk reduction strategies among in-school youth [29], [33], [40], [41], [55]–[60]. The remaining studies provided comprehensive sex education.

Many studies reported using a variety of instructional formats to convey information and generate discussion. For example, 35 studies used lectures, 34 employed interactive discussions, 30 incorporated role-plays, and 21 utilized skill-based sessions, such as learning the steps involved in correct condom use. Use of media such as videos and audio tapes was also common. Two studies relied on drama, including creating plays and skits, for the bulk of the intervention [61], [62] and one study used a comic book to impart information [63]. Two studies were internet-based [36], [64] and involved students completing online modules during school hours at their own pace. Thirty three interventions reported basing their intervention on theory. Theories commonly referenced were Social Cognitive Theory, Health Belief Model, and Theory of Reasoned Action.

Intervention content varied widely. Several interventions were adapted from curricula that had been developed and implemented elsewhere, such as the US-based “Focus on Kids” [31], [38], [43], “Making Proud Choices” [41], and WHO's “Responsible Behavior: Delaying Sex” [40]. Other interventions worked with local community members and health educators to develop appropriate curricula. Forty-one studies included some form of commnity involvement in the intervention, such as consulting with parents and communities about what content could and could not be included in the intervention [33], [55], [56], [58], [60], [65]–[68]. Many studies reported a reluctance by teachers, parents, and/or communities to discuss or allow discussion of condoms during the intervention [33], [55], [56], [65], [66], [68]. Interventions commonly addressed health-related issues in addition to HIV/AIDS prevention (n = 43), including reproductive biology (n = 22), pregnancy prevention (n = 16), STI prevention (n = 22), relationships (n = 10), violence (n = 5), and values clarification (n = 5).

#### Intervention duration, location, and instructor type

Interventions ranged from 2 or 3 hours in total [69], [70] to spanning multiple years and involving community-based components such as the creation of a youth resource center, youth-led condom distribution [30], [37], and training healthcare workers how to provide youth-friendly health information [68]. Most studies took place during the regular school day, although several studies included alternate times for the school-based sex education, such as having sessions on Saturdays [40] or after school hours [31], [38], [60]. Interventions were facilitated by health professionals, teachers, or peer educators, although several studies involved a mix of facilitator types throughout the intervention. The majority of studies took place in urban (n = 34) or peri-urban settings (n = 2). Only 3 studies specified that interventions took place in rural areas, 14 studies were conducted in a mix of urban and rural settings, and 11 studies did not provide enough information to determine the type of setting in which the intervention took place.

### Meta-analysis results

#### HIV/AIDS knowledge

HIV-related knowledge was the most commonly reported study outcome. Of all included studies, 49 (76.6%) evaluated the effect of the intervention on HIV-related knowledge. The measures used to assess HIV-related knowledge varied by study but centered on aspects such as HIV biology, HIV transmission, and HIV prevention. Some studies measuring HIV-related knowledge were excluded from meta-analysis for not controlling for baseline differences in socio-demographics or outcome variables (n = 3) [29], [71], [72], not providing enough data to convert results into standardized effect sizes (n = 18) [36], [47]–[50], [56], [57], [62], [63], [65], [69], [73]–[78], presenting knowledge outcomes not specifically related to HIV (n = 2), such as asking students whether they knew about HIV/AIDS [58], and measuring how many STIs students correctly identified [66].

**Figure 2:**
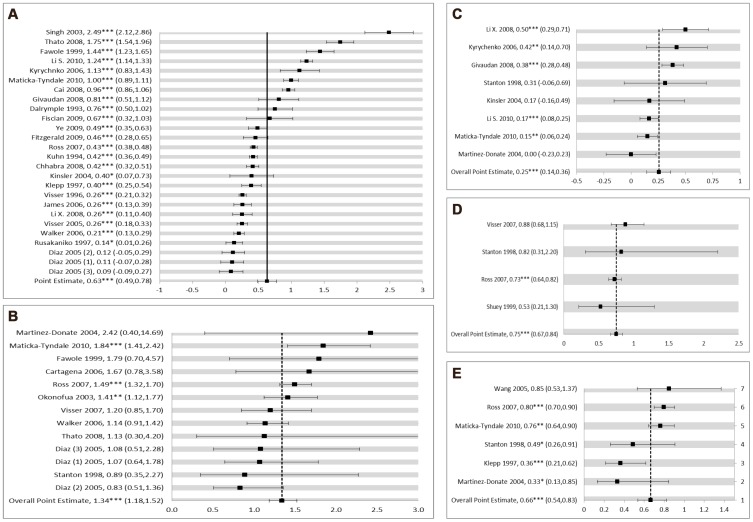
Forest plots from meta-analysis of (A) HIV-related knowledge, (B) condom use, (C) self-efficacy, (D) number of sexual partners, and (E) initiation of sex. A: Effect of school-based sex education on HIV-related knowledge (random effects, Hedges' G). B: Effect of school-based sex education on condom use (random effects, odds ratio). C: Effect of school-based sex education on self-efficacy (random effects, Hedges' G). D: Effect of school-based sex education on number of sexual partners (random effects, odds ratio). E: Effect of school-based sex education on initiation of first sex (random effects, odds ratio). *Note*. Results from Diaz et al., 2005, Fiscian et al., 2009, Givauden et al., 2008, Klepp et al., 1997, Visser et al., 1996, Visser et al., 2005, Cartagena et al., 2006, Okonofua et al., 2003, and Li et al., 2008 were adjusted for baseline differences and certain covariates. Values from remaining studies are unadjusted. *p<0.05, **p<0.010, and ***p<0.001.

Twenty-six studies were included in the meta-analysis for knowledge; results are presented in Table 3 and Figure 2. Nineteen studies reported knowledge as a continuous outcome; seven reported it dichotomously. In meta-analysis, dichotomous outcomes were converted to standardized mean differences for comparison across all studies. Random effects meta-analysis suggests that students who were exposed to a sexual education intervention were more knowledgeable of HIV and related topics than youth who did not experience an intervention. The standardized mean difference (Hedges' *g*) was 0.63 with a 95% confidence interval of 0.49 to 0.78, p<0.001. Meta-regression indicated no significant standardized mean difference when comparing males and females (p = 0.194); however, only 3 studies reported data disaggregated by gender with two additional studies reporting on entirely female samples. Similarly there was no significant difference in standardized mean knowledge score differences comparing abstinence-focused to comprehensive sex education interventions (p = 0.501). When stratified by instructor type, interventions led by health professionals (e.g. doctors, nurses, or health educators), appeared to produce more knowledgeable students than those led by either teachers, peers, or a mix of different types of instructors (β = 0.65, p = 0.004). However, when the two interventions implemented among college students were removed [42], [43], this effect was no longer significant.

**Table 3:** Meta-analytic outcomes assessing associations between school-based sex education interventions and HIV/AIDS knowledge, condom use, initiation of first sex, number of sex partners, and self-efficacy.

Outcome	*k*	Point Estimate (ES)^a^	*SE*(ES)^b^	*β* c	*SE*(*β*)	*I^2^*	95% CI(*I^2^*)
**HIV/AIDS Knowledge** d							
Overall	26	0.63***	.07			97.6	97.1–98.0
By Instructor Type	26	0.63***	.07				
Health	3	1.22***	.20	0.65**	.07		
Mix	10	0.46***	.11	−0.27	.16		
Peer	5	0.71***	.15	0.10	.17		
Teacher Only	8	0.59***	.11	−0.06	.16		
By Follow-up Length	26	0.63***	.07				
≤3 Months	8	0.70***	.14	0.09	.17		
3 to 11 Months	8	0.87***	.14	0.34	.15		
≥12 Months	10	0.41***	.12	−0.38*	.16		
By Age	26	0.64***	.08				
Younger (≤15)	7	0.58***	.48	−0.21	.34		
Older (>15)	9	0.98***	.13	1.12***	.34		
Wide Range	10	0.37**	.12	−0.87**	.31		
**Condom use** e							
Overall	12	1.34***	.06			29.2	0–63.4
**Initiation of first sex** e							
Overall	6	0.66***	.11			62.1	7.6–84.4
**Number of sex partners** e							
Overall	4	0.75***	.06			0	0–83.4
**Self-efficacy** d							
Overall	8	0.25***	.06			73.2	45.4–86.9

*Note*. *K* = number of studies.

a Hedges' *g* when continuous, odds ratio when dichotomous.

b Standard error of log odds ratio when outcome is dichotomous.

c Contrast between point estimate of given variable category and combined point estimate of remaining categories.

d Continuous measure.

e Dichotomous measure.

*p<.05,

**p<.01,

***p<.001.

When considering length of follow-up time, standardized mean differences in knowledge scores were largest when data were collected 3 to 11 months post-intervention compared to all other categories of follow-up time (Hedges' *g* = 0.87, 95% CI 0.60–1.13, p<0.001). The smallest differences were seen when knowledge was measured 3 months or less post-intervention (Hedges' *g* = 0.70, 95% CI: 0.43–0.97, p<0.001). Studies with the shortest follow-up time generally corresponded to studies of the shortest duration (≤3 weeks). However, duration was difficult to measure due to wide variations in length and intensity of intervention implementation. Two studies evaluated the long-term effects (≥3 years post-intervention) of year-long interventions based in schools in Tanzania [30] and Kenya [68]. The study from Tanzania found that effects on knowledge remained significant after 3 years, but limited sustained effects on behaviors were seen [30]. The study from Kenya found significant improvements in HIV-related knowledge 3 years post-intervention, although knowledge initially appeared to decline in the period from pre-intervention to 18-months [68]. Regarding type of measure used, studies measuring knowledge as a dichotomous outcome generated smaller effect sizes than studies measuring knowledge continuously; this difference was statistically significant (β = −0.42, p = 0.02).

A funnel plot of the distribution of effect sizes by their standard error (figure not shown) suggests that some bias exists across studies as the plot was asymmetrical, indicating a possible underdispersion of less precise effect sizes. Following the assumption of normally distributed effect sizes across studies, finding more studies demonstrating at least a null effect would be expected, but finding studies showing a negative effect is less likely given the nature of the outcome, knowledge. Given the wide range of scales used to assess knowledge and the different facets of sexual education being taught across interventions, the asymmetrical plot could also be interpreted as presenting more evidence of a truly heterogeneous effect of school-based sexual education on HIV-related knowledge. The I^2^ value also supports this hypothesis.

#### Self-efficacy

Twenty-two studies measured self-efficacy. Fourteen of these studies were excluded from meta-analysis for measuring general self-efficacy or self-efficacy not related to condom use or sex refusal (n = 8) [41], [46], [51], [64], [71], [79], [80] or for not providing enough data to convert to standardized effect size measures (n = 6) [50], [62], [73], [74], [77], [78]. The remaining eight studies measured self-efficacy related to either sex refusal or condom use and were included in meta-analysis [31], [43], [68], [81]–[85]. All but one study included in meta-analysis measured self-efficacy on a continuous scale; the remaining study measured self-efficacy as a proportion. The one dichotomous outcome was transformed into a standardized mean difference to allow comparison across studies. The standardized mean difference (Hedges' g) comparing those who received the intervention to those who did not was 0.25 (95% CI: 0.14–0.36, p<0.001), meaning those receiving the intervention exhibited significantly more self-efficacy in regards to being able to refuse sex or use a condom during sex. The intervention with the largest mean difference in self-efficacy was implemented among university students in Nanjing, China using an adapted version of the “Focus on Kids” program originally identified as an effective intervention by the U.S. Centers for Disease Control and Prevention (CDC), which involved group activities and games facilitated by trained graduate students and faculty [43]. The intervention with the second highest mean difference in self-efficacy involved an intervention delivered to secondary students in Ukraine by trained young physicians consisting of 6 weekly 1 hour sessions focusing on HIV transmission and biology, condom use negotiation skills, preventive measures, and dangers of drug use through small group activities, role playing, games, and discussions [83]. This intervention was adapted using the WHO Training and Resource Manual on School Health and HIV Prevention. One included study involving a 3 hour HIV-prevention workshop found no increase in condom use self-efficacy [85].

#### Condom use

Twenty one studies measured condom use as a primary outcome. Of these, 13 were eligible for meta-analysis [31], [37], [52], [59], [66], [68], [73], [85]–[88]. Eight studies were excluded for not presenting enough data to convert to a standardized effect size [39], [43], [45], [62], [77], [78], [82], [89]. Condom measures included in the analysis were condom use at last sex, 100% condom use, and consistent condom use (all dichotomous measures). When synthesized across interventions, condom use was significantly higher among intervention participants (OR = 1.34, 95% CI: 1.18–1.52, p<0.001). Individually, only three of the twelve studies found a significant difference in condom use between intervention and control groups [37], [66], [68]. All three of these studies included some form of training for healthcare workers outside of the school-setting on how to provide youth-friendly sexual and reproductive health information, including condom use [37], [66], [68]. In one study based in Kenya, providing lessons on condom use was met with strong resistance from teachers who feared that teaching students about condoms would encourage sexual activity [68]. As a result, condom use was not included in the regular lesson plans although teachers were trained on how to respond to students' questions about condoms in a factual manner [68]. Of all interventions included in this meta-analysis, there was one abstinence-plus program that reported emphasizing delay of sexual debut until marriage while still provided information on other prevention measures, such as condom use [59]. This study did not find a significant difference in consistent condom use comparing those who received the intervention to those who did not 3-months post-intervention (OR = 1.13, 95% CI: 0.30–4.20, p = 0.86).

#### Number of sex partners

Of 10 studies measuring participants' number of sexual partners, four were eligible for meta-analysis [31], [37], [58], [87]. Six were excluded for not containing enough data to convert results into standardized effect sizes [36], [45], [59], [62], [78], [90]. Of the four studies included in meta-analysis, three presented results dichotomously; the remaining study presented results continuously (mean number of sex partners). The continuous outcome was converted to a standardized mean difference to allow for comparison across all studies. Outcomes synthesized for this analysis included: having greater than 1 partner in the last 12 months [37], mean reported number of partners [58], having greater than 2 sexual partners in the last 6 months [31], and reporting multiple partners during the past 3 months [87]. Across studies students receiving interventions demonstrated a 25% reduction in odds of reporting more partners compared to control or comparison groups (OR = 0.75, 95% CI: 0.67–0.84, p<0.001). However, individually, only one study with a large sample size (n = 6877) showed students receiving the intervention reported fewer sex partners [37] whereas the other studies showed a non-significant difference. Data from this study were collected 36 months following the intervention, which was the longest reported follow-up of all included studies [37].

#### Initiation of first sex

Nine studies measured initiation of first sex. Three were excluded for not containing enough data to convert results into standardized effect sizes for meta-analysis [62], [78], [90]. The remaining six studies were included in meta-analysis [31], [33], [37], [60], [68], [85]. All studies measured this outcome dichotomously. Participants who received the intervention had a 34% reduction in odds of initiating sexual intercourse during follow-up compared to control or comparison groups (OR = 0.66, 95% CI = 0.54–0.83, p<0.001). Individually all but one study [60] showed significant reductions in sexual debut for those who received the intervention. The study showing the highest reduction in sexual debut involved activities that took place beyond the classroom setting, including the provision of youth friendly reproductive health services, condom distribution, and community mobilization [37]. The one abstinence-only program that measured sexual initiation [33] also showed a significant reduction in odds of sexual debut between the 6^th^ and 7^th^ grade school years for youth who received the intervention (OR = 0.36, 95% CI 0.21–0.62, p<0.001).

## Discussion

This review found that school-based sex education is an effective intervention for generating HIV-related knowledge and decreasing sexual risk behaviors among participants, including delaying sexual debut, increasing condom use, and decreasing numbers of sexual partners. Importantly, no individual study included in meta-analysis, including abstinence-only, abstinence-plus, and comprehensive school-based sex education interventions, found detrimental effects of school-based sex education on increased risky sexual behavior. This finding is notable given that some argue programs including information on abstinence *and* safe sex strategies give mixed messages to students and may promote sexual activity [91].

Comprehensive school-based sex education comprised the majority of interventions included in this review despite extensive attempts to identify abstinence-only and abstinence-plus interventions. Given PEPFAR's past emphasis on abstinence-only and abstinence-plus interventions [4], it is surprising to find so few peer reviewed evaluations of this strategy that met our inclusion criteria. The uneven distribution in comprehensive versus abstinence-only or abstinence-plus interventions made it difficult to compare the effectiveness of these intervention types. Additionally, many abstinence-only or abstinence-plus interventions measured outcomes related to HIV-knowledge but did not include outcomes related to sexual risk behavior, such as condom use or number of sexual partners, thus rendering comparisons to comprehensive sex education unfeasible. These findings are similar to those of parallel systematic reviews and meta-analyses comparing the effectiveness of comprehensive sex education and abstinence-only interventions, which found that although comprehensive sex education interventions were effective at reducing high-risk sexual behavior, no conclusion could be drawn from interventions emphasizing abstinence due to the small number of eligible studies and inconsistent findings [10].

Interventions producing the most significant changes in behavior seemed to have several characteristics in common. First, several effective interventions included community-based components that extended beyond school-based sex education by involving resources and activities outside of the school environment, such as training healthcare staff to offer youth-friendly services, distributing condoms, and involving parents, teachers, and community members in intervention development. Additionally, studies that adapted curricula from interventions already deemed efficacious also tended to produce significant changes on HIV-related behaviors. These findings are similar to another review which concluded that replicating effective sex education programs continued to produce significant behavior changes even when programs were implemented in different settings [92].

It is encouraging that many of the reviewed studies used controlled designs and randomized intervention assignments, especially as earlier reviews have emphasized the need to improve study designs when evaluating school-based sex education interventions [92], [93]. Despite this improvement, many studies exhibited methodological flaws that have been discussed at length elsewhere [94], which inhibited our ability to include these studies in meta-analysis. Out of 64 included studies, 50 measured at least one of the five outcomes included in this meta-analysis, and of these 33 provided enough data to include in the quantitative synthesis. This finding highlights the need to further improve the methodological rigor of studies.

### Limitations

This review must be seen in light of several limitations. Firstly, all outcomes reported in this review were based on self-report, which creates potential for social desirability bias and memory error. Secondly, we combined outcomes in meta-analysis that are not identical, such as combining effect sizes generated from different scales measuring HIV-related knowledge, which could lead to inaccuracies in synthesized effects. We also combined both continuous and dichotomous outcomes in several meta-analyses, which could introduce error [95], although comparability has been shown between continuous and dichotomous outcomes used in meta-analysis when certain assumptions are met [96]. Additionally, it is possible our search strategy excluded potentially eligible articles. We used multiple search strategies in an effort to minimize this risk. This review could also be affected by publication bias, which was assessed by constructing funnel plots when feasible, although several studies showing null effects for various outcomes were included.

### Conclusions

School-based sex education is a critical tool for HIV prevention among youth, and research suggests school-based HIV prevention programs are cost-effective when implemented in the context of combination prevention [97]. Intervention evaluations need to go beyond addressing the question of whether school-based sex education increases knowledge and focus instead on understanding implementation factors that led to the most success in shaping and changing subsequent HIV-related risk behaviors. As recently stated by the United Nations Special Rapporteur on the Right to Education, access to education about sex and reproductive health is a human right [98]; therefore greater efforts should be made to identify and scale-up effective interventions. However, school-based education alone cannot be relied on to prevent HIV infections among young people since not all young people attend school and since school funds and resources are often already strained. Instead, school-based sex education should be part of more holistic HIV prevention intervention aiming to engage young people in learning about and shaping their sexual and reproductive future.

## Supporting Information

Checklist S1 **PRISMA Checklist for systematic reviews.**
(DOC)
